# The liver fibrosis index is superior to the APRI and FIB-4 for predicting liver fibrosis in chronic hepatitis B patients in China

**DOI:** 10.1186/s12879-019-4459-4

**Published:** 2019-10-22

**Authors:** Dedong Huang, Taofa Lin, Shaoyang Wang, Lieyun Cheng, Liping Xie, Youguang Lu, Muxing Chen, Lingling Zhu, Jie Shi

**Affiliations:** 1 Department of Infectious Diseases, the 903rd Hospital of PLA, Hangzhou, China; 2 Department of Infectious Diseases, the 900th Hospital of PLA, No.156 North Road West 2nd Ring Road, Fuzhou, 350013 China; 3 Clinical educational Institute of the 900th Hospital of PLA affiliated Fujian Medical University, No.156 North Road West 2nd Ring Road, Fuzhou, 350013 China; 4 Department of ultrasound, the 900th Hospital of PLA, Fuzhou, China

**Keywords:** Hepatitis B, Laboratory examinations, Liver fibrosis, Ultrasound elastography, APRI, FIB-4

## Abstract

**Background:**

The purpose of this study was to prospectively investigate the value of real-time ultrasound elastography (RTE) for the diagnosis of liver fibrosis (LF) in patients with chronic hepatitis B (CHB), to correlate the elastography findings with the histologic stage of LF and to compare RTE findings with those from noninvasive tests of LF calculated using laboratory blood parameters.

**Methods:**

Liver biopsies, laboratory blood testing, and RTE were performed in 91 patients with CHB. The LF index (LFI) was calculated using a multiple linear regression equation involving 11 parameters, which represented the degree of LF. The higher the LFI is, the greater the degree of LF.

**Results:**

The mean aspartate aminotransferase-to-platelet ratio index (APRI) and the mean fibrosis index based on four factors (FIB-4) were significantly different for the 5 stages of LF, respectively. The APRI (*r* = 0.43, *P* = 0.006), FIB-4 (*r* = 0.51, *P* = 0.012) and LFI (*r* = 0.562, *P* = 0.004) were correlated with the stages of LF. For discriminating stage F0 from F1, only the LFI had significant power (*P* = 0.026) for predicting stage F1. For discriminating stage F4 from F3, only the LFI had statistically significant power (*P* = 0.024) in predicting stage F4. The areas under the receiver operating characteristic curves (AUCs) of the LFI for diagnosing significant, advanced LF and liver cirrhosis were significantly higher than those of the APRI and FIB-4, and the LFI had better sensitivity and specificity.

**Conclusions:**

The LFI calculated by RTE is reliable for the assessment of LF in patients with CHB and has better discrimination power than the APRI and FIB-4.

## Background

Hepatitis B virus (HBV) infection remains a severe global public hygiene and clinical problem: approximately 240 million people have HBV in the world. People infected with HBV have increased risk of developing liver fibrosis or cirrhosis, hepatic decompensation, and hepatocellular carcinoma (HCC) [1]. Liver fibrosis (LF) is important in determining whether, when and how to initiate antiviral therapy. The degree of LF or cirrhosis is an independent factor to predict the mortality in chronic hepatitis B (CHB) patients [2]. The 1-year mortality rates decreased from 57% in those with severe cirrhosis to 1% in patients with early-stage LF. Moreover, about 10–17% liver cirrhosis patient will develop HCC in 5 years [2]. Early accurate assessment of LF in patients infected with HBV is essential not only for the better outcomes associated with early initiation of antiviral treatment, but also for predicting the long-term clinical prognosis [2, 3].

At present, the gold standard for the diagnosis of LF or cirrhosis is liver biopsy [2, 4]. However, liver biopsy was an invasive technique that maybe induce the patient physical or/and mental discomfort, complications and contraindications, which restricted it widespread utilization in routine practice. Sometimes, sampling errors may affect the accuracy of liver biopsy. Furthermore, intraobserver and interobserver discrepancies may induce bias in staging LF when analysing the same liver biopsy sample [2, 4, 5]. Therefore, a few alternative noninvasive methods have been developed intensely and have improved evaluation of the LF stage, such as fibrosis index based on four factors (FIB-4), sonographic transient elastography (Fibroscan), the aspartate aminotransferase-to-platelet ratio index (APRI) and real-time tissue elastography [3, 5–10]. As noninvasive methods, the APRI and FIB-4 have been recommended to determine the stage of LF in resource-limited countries by the WHO guidelines and many other guidelines [1, 10–13].

Fibroscan, recently reported by many studies, is a noninvasive device that can be used to grade the stage of liver fibrosis or cirrhosis [3, 5, 9, 14]. Fibroscan can not only predict cirrhosis-linked complications in patients with HBV, but also forecast the recurrence of HCC after curative resection. However, Fibroscan may be difficult to detect liver stiffness in obese patients, in narrow intercostal spaces patients and in ascites patients [14–18]. Fibroscan is still expensive and it can only be used in main hospitals in some big cities in China. Thus, it’s urgent to found a simple, cheap and noninvasive LF or cirrhosis detection system in China.

Real-time ultrasound elastography (RTE) is a new sonography-based noninvasive technique for assessing liver tissue elasticity. RTE detects the propagation speed of shear waves for assessing LF or cirrhosis, which is technically different from Fibroscan [6, 19–23]. RTE can capture the 2D strain images induced by internal heartbeats. These strain images show that more patchiness is, the higher degree LF or cirrhosis is [24]. Therefore, RTE may be utilized in the ascites patients or obese patients.

In recent, RTE has been reported to be effective in chronic hepatitis C patients [25]. However, to date there are only a few available reports on the diagnostic efficacy of RTE for measuring LF or cirrhosis in people with HBV in China. The aims of this study were to assess the accuracy of the quantitative measurement of LF in patients with HBV by RTE, to determine the LF fibrosis index (LFI) for different stages of LF and to compare the accuracy among the LFI, the APRI, and the FIB-4 for grading the stages of LF in patients using liver biopsy as the reference standard.

## Methods

### Patients

Ninety-one patients who underwent liver biopsy for grading of liver fibrosis at the 900th Hospital of PLA from January 2014 to June 2018 were recruited. All patients were diagnosed as CHB, according to Asian-Pacific clinical practice guidelines about CHB patients management [1]. HBV Markers (HBsAg, anti-HBs, HBeAg, anti-HBe and anti-HBc) were detected by an i2000 immunoassay instrument (Abbott Laboratories). The kits [HBsAg (Cat. No. 6C36–32), anti-HBs (Cat. No. 7C18–30), HBeAg (Cat. No. 6C32–20), anti-HBe (Cat. No. 6C34–20) and anti-HBc (Cat. No. 8 L44–30)] were acquired from commercial way. All CHB patients were already starting regular followed up visits in the hospital and underwent laboratory investigations and diagnostic liver biopsy for identification of their fibrosis stage before starting or declining antiviral treatment. In all patients, the LFI was measured by RTE and blood samples were taken for Laboratory examinations within 24 h before liver biopsy. This study was approved by Ethics Committee of the 900th Hospital of PLA and the Institutional Subcommittee. The document of institutional review board approval and written informed patient consent from each participant were all obtained. The exclusion criteria of this study were as follows: patients with other types of hepatitis, patients with metabolic disease, patients with liver disease associated with drugs, patients with alcoholic liver disease, patients with HIV and patients with cardiopulmonary disease. The demographic and clinical parameters of every patient, such as age, sex, APRI, FIB-4, were shown in Table 1.

**Table 1:** Main demographic and laboratory characteristics of the patients

Characteristics	Total (*n* = 91)	
Sex (*n*, %)		
Male	49	53.85%
Female	42	46.15%
Age (year)	41.05	± 11.97
ALT (IU/L)^a^	86.31	± 62.69
AST (IU/L)^a^	46.04	± 31.98
AST/ALT^a^	1.04	± 0.53
GGT (IU/L)^a^	91.39	± 82.44
PLT (10^9^/L)^a^	172.21	± 72.58
APRI^a^	1.40	± 0.96
FIB-4^a^	6.70	± 2.14
LFI^a^	3.18	± 0.84
Stage of fibrosis (*n*, %)		
F0	12	13.19%
F1	18	19.78%
F2	21	23.08%
F3	19	20.88%
F4	21	23.08%

^a^Mean ± standard deviation

*ALT* alanine aminotransferase, *AST* aspartate aminotransferase, *GGT* gamma-glutamyl transpeptidase, *PLT* platelet count, *APRI* aspartate aminotransferase-to-platelet ratio index, *FIB-4* fibrosis index based on four factors, *LFI* liver fibrosis index

### Liver histologic analysis

According to liver ultrasound examination, percutaneous liver penetration was performed using an 18-gauge Tru-Cut™ needle (Medical Technology, Gainesville, FL, USA), and samples were taken from liver tissue under the right intercostal of each patient [3]. The biopsy specimen was at least 1.2 cm length and had at least 6 portal tracts [4, 7, 17]. The liver biopsy specimens were rapidly fixed in 4% buffered formalin and then embedded in paraffin block. The liver specimen was cut into four-micrometre-thick sections and were stained with haematoxylin-eosin and Masson’s trichrome. An experienced pathologist who was blinded to all patient’s data scored the liver biopsy specimens from F0 to F4. The LF stage was assessed according to the METAVIR and classified as follows: F0, no fibrosis; F1, portal fibrosis without septa; F2, portal fibrosis and a few septa; F3, numerous septa without cirrhosis; and F4, cirrhosis [26].

### Laboratory analysis and determination of the APRI and FIB-4

Complete venous blood samples were taken from every participant with empty stomach within 24 h before elastography. Including platelet count (PLT), routine blood tests were analysed. The following liver biochemistry parameters were determined: aspartate aminotransferase (AST), alanine aminotransferase (ALT), γ-glutamyl transpeptidase (γ-GGT), total cholesterol, AST to platelet ratio index. The APRI and FIB-4 were obtained by the following formulas: [3, 9, 14, 15].

$$ \mathrm{APRI}=\frac{\left(\left(\mathrm{AST}\left(\mathrm{IU}/\mathrm{L}\right)\right)/\left(\mathrm{UL}{\mathrm{N}}^{\ast}\mathrm{ofAST}\left(\mathrm{IU}/\mathrm{L}\right)\right)\right)}{\mathrm{PLT}\left({10}^9/\mathrm{L}\right)}\times 100 $$

$$ \mathrm{FIB}-4=\frac{\mathrm{Age}\left(\mathrm{years}\right)\times \mathrm{AST}\left(\mathrm{IU}/\mathrm{L}\right)}{\mathrm{PLT}\ \left({10}^9/\mathrm{L}\right)\times \sqrt{\mathrm{ALT}\left(\mathrm{IU}/\mathrm{L}\right)}}\times 100 $$

### Real-time tissue elastography

Elasticity of liver tissue was detected with real-time tissue elastography by an experienced physician, according to the protocol and literature [4, 19–22, 24, 25, 27]. The physician was blinded to all laboratory data. Real-time tissue elastography was measured with an HI VISION 900 ultrasound device (Hitachi Medical Systems Co. Ltd., Tokyo, Japan) using a 3–7-MHz linear array probe within 24 h before liver biopsy. All patients were examined in a facilitate supine position with the right arm extended above the head. The physician, using slight manual compression, pressed the linear probe at the right liver through an intercostal space. The equipment can automatically correct the internal distortion of the liver tissue caused by heart beat while the participants briefly held their breath. To obtain good images, scans avoided large vessels, the lungs and the ribs. A 30 mm in length, 20 mm in width and 10 mm below the liver capsule rectangular area was set as the region of interest (ROI) for all participants. While the colour-coded images on real-time tissue elastography were stable, the frame of the ROI was set. The eleven parameters associated with tissue stiffness in the ROI shown by the ultrasound device were acquired automatically. These paraments included the low-strain (blue) area ratio within the ROI, the mean and standard deviation of the relative strain within the ROI, the skewness and kurtosis on the strain histogram, the textural complexity, the textural homogeneity (angular second moment), the complexity of the low-strain region, the textural local homogeneity (inverse difference moment), contrast, and the correlation. The LFI was calculated using a multiple linear regression equation including eleven parameters, which represented the stage of LF, as described before [9, 19, 20, 24]. The higher the LFI was, the greater the LF stage was.

### Statistical analysis

Statistical analyses were performed using SPSS version 18.0 (SPSS Inc., Chicago, IL, USA). Quantitative data are expressed as the means ± standard deviation (M ± SD), while qualitative data are displayed as numbers and percentages. The *t*-test or Mann–Whitney U test was used to compare continuous variables between two groups*.* Comparative analyses of more than two groups were performed using analysis of variance (ANOVA). The correlations of ordinal categorical variables were analysed by Spearman’s rank correlation coefficient analysis. Significant differences were assessed by the chi-squared test and Fisher’s exact test for categorical variables. The correlations of differences were considered statistically significant at *P* < 0.050. The diagnostic performances of the LFI, APRI, and FIB-4 were assessed by receiver operating characteristic (ROC) curves. The areas under the ROC curves (AUCs) were calculated with 95% confidence intervals. The cut-off values of different noninvasive methods on different LF stages were determined by You Den Index. The cut-off values were chosen at maximizing the sensitivity and specificity and diagnostic accuracy.

## Results

### Baseline patient characteristics

The main demographic and laboratory characteristics of the study patients are presented in Table 1. A total of 91 patients were enrolled, of whom 53.85% (49) were male, and 46.15% (42) were female, with a mean age 41.05 ± 11.97 years. The mean ALT, AST, AST/ALT, γ-GGT and PLT were 86.31 ± 62.69 IU/L, 46.04 ± 31.98 IU/L, 1.04 ± 0.53, 91.39 ± 82.44 IU/L and 172.21 ± 72.58 × 10^9^/L, respectively. The means APRI, FIB-4, and LFI were 1.40 ± 0.96, 6.70 ± 2.14 and 3.18 ± 0.84, respectively. According to the METAVIR score, the number of patients in the F0, F1, F2, F3 and F4 stage was 12 (13.19%), 18 (19.78%), 21 (23.08%), 19 (20.88%) and 21 (23.08%).

### Comparison of the APRI, FIB-4 and laboratory characteristics in each stage of fibrosis

**Fig. 1:**
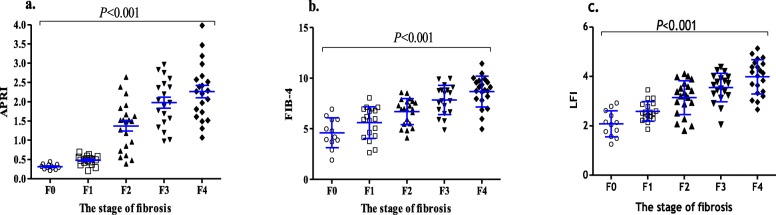
Comparison of the APRI, FIB-4 and LFI according to the stage of fibrosis. **a** the APRI for each stage, **b** the FIB-4 for each stage of fibrosis, **c** LFI for each stage of fibrosis. APRI: aspartate aminotransferase-to-platelet ratio index, FIB-4: fibrosis index based on four factors, LFI: liver fibrosis index

Age and γ-GGT increased significantly with an increasing severity of fibrosis (*P* = 0.018 and *P* = 0.006), while the PLT decreased significantly with increasing severity of fibrosis (*P* < 0.001). The gender ratio at each stage showed a statistically nonsignificant difference. There was no significant difference in AST and ALT according to the stage of fibrosis (Table 2). The mean of APRI in the 5 fibrosis stages was 0.32 ± 0.07, 0.48 ± 0.10, 1.37 ± 0.63, 1.98 ± 0.62 and 2.26 ± 0.73, respectively. The mean of FIB-4 in the 5 fibrosis stages was 4.61 ± 1.47, 5.61 ± 1.57, 6.69 ± 1.29, 7.84 ± 1.45 and 8.67 ± 1.52, respectively. The mean LFI in the 5 fibrosis stages was 2.19 ± 0.48, 2.54 ± 0.36, 3.14 ± 0.68, 3.56 ± 0.54 and 4.00 ± 0.66, respectively (Fig. 1). The APRI, FIB-4 and LFI in the 5 fibrosis stages were significantly different (*P* < 0.001). These indexes also showed increasing trends with fibrosis stages in CHB patients. The APRI (*r* = 0.43, *P* = 0.006), FIB-4 (*r* = 0.51, *P* = 0.012) and LFI (*r* = 0.562, *P* = 0.004) were correlated with the stage of LF, according to Spearman’s rank correlation coefficient analysis.

**Table 2:** Patient characteristics according to the Scheuer fibrosis stage

Characteristic	F0(*n* = 12)^*^	F1(*n* = 18)	F2(*n* = 21)	F3(*n* = 19)	F4(*n* = 21)	*P*
Age (year)	32.25 ± 10.36	38.17 ± 10.99	40.90 ± 11.45	43.68 ± 12.37	46.33 ± 13.46	0.018
Male/female^§^	7/5	9/9	12/9	10/9	11/10	0.988^#^
ALT (IU/L)	60.67 ± 17.84	83.22 ± 54.43	83.30 ± 57.13	94.21 ± 74.63	105.35 ± 75.36	0.345
AST (IU/L)	35.58 ± 16.22	35.08 ± 21.16	41.46 ± 26.34	52.58 ± 38.82	60.18 ± 39.41	0.066
GGT (IU/L)	46.50 ± 32.95	55.53 ± 38.34	90.49 ± 53.86	104.32 ± 99.57	136.96 ± 110.49	0.006
PLT (10^9^/L)	254.08 ± 72.08	194.50 ± 50.08	182.76 ± 47.16	159.42 ± 79.28	107.33 ± 40.92	< 0.001

Data are presented as the mean ± standard deviation and analysed by analysis of variance (ANOVA) unless indicated. *ALT* alanine aminotransferase, *AST* aspartate aminotransferase, *GGT* gamma-glutamyl transpeptidase, *PLT* platelet count, *APRI* aspartate aminotransferase-to-platelet ratio index, *FIB-4* fibrosis index based on four factors, *LFI* liver fibrosis index. ^*^Number in each fibrosis stage. ^§^Number of males and females. ^#^Chi-square test

Figure 1 Comparison of the APRI, FIB-4 and LFI according to the stage of fibrosis. Figure 1a the APRI for each stage, 1b. the FIB-4 for each stage of fibrosis, 1c. LFI for each stage of fibrosis. APRI: aspartate aminotransferase-to-platelet ratio index, FIB-4: fibrosis index based on four factors, LFI: liver fibrosis index

### Relationship of the APRI, FIB-4, and LFI to the stage of LF

**Fig. 2:**
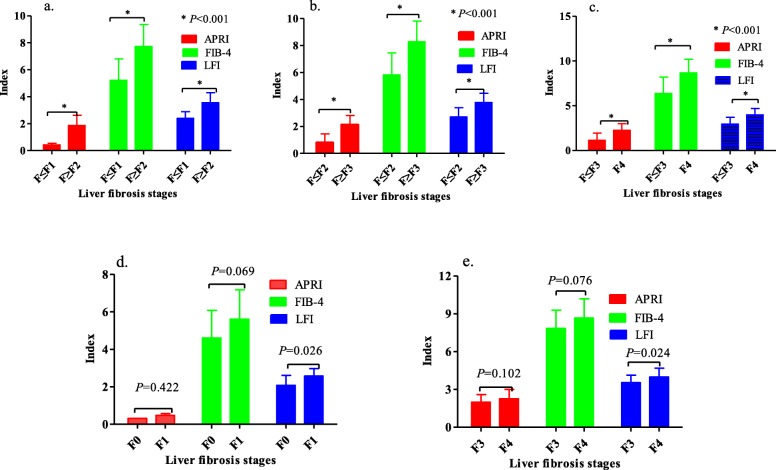
Relationship of the APRI, FIB-4, and LFI to LF stages. **a** Comparison of the APRI, FIB-4 and LFI for discriminating significant LF (F ≥ F2); **b** Comparison of the APRI, FIB-4 and LFI for predicting advanced liver cirrhosis (F ≥ F3); **c** Comparison of the APRI, FIB-4 and LFI for diagnosing liver cirrhosis. **d** Comparison of the APRI and FIB-4 with the LFI for discriminating stage F1 from F0; **e** Comparison of the APRI and FIB-4 with the LFI for discriminating stage F4 from F3. APRI: aspartate aminotransferase-to-platelet ratio index, FIB-4: fibrosis index based on four factors, LFI: liver fibrosis index

The APRI, FIB-4, and LFI in the patients with significant LF (F ≥ F2) were significantly higher than in those with an LF stage ≤F1 (Fig. 2a). The APRI, FIB-4, and LFI in patients with advanced LF (F ≥ F3) were significantly higher than in those with an LF stage ≤F3 (Fig. 2b). The APRI, FIB-4, and LFI in the patients with cirrhosis (F=F4) were significantly higher than in those with no liver cirrhosis (F ≤ F3) (Fig. 2c). However, for discriminating stage F0 from F1, only the LFI had significant power (*P* = 0.026) for predicting stage F1. For discriminating stages F4 and F3, only the LFI had a statistically significant power (*P* = 0.024) for predicting stage F4.

### Comparisons of AUCs of the APRI, FIB-4, and LFI for various stages of LF

**Fig. 3:**
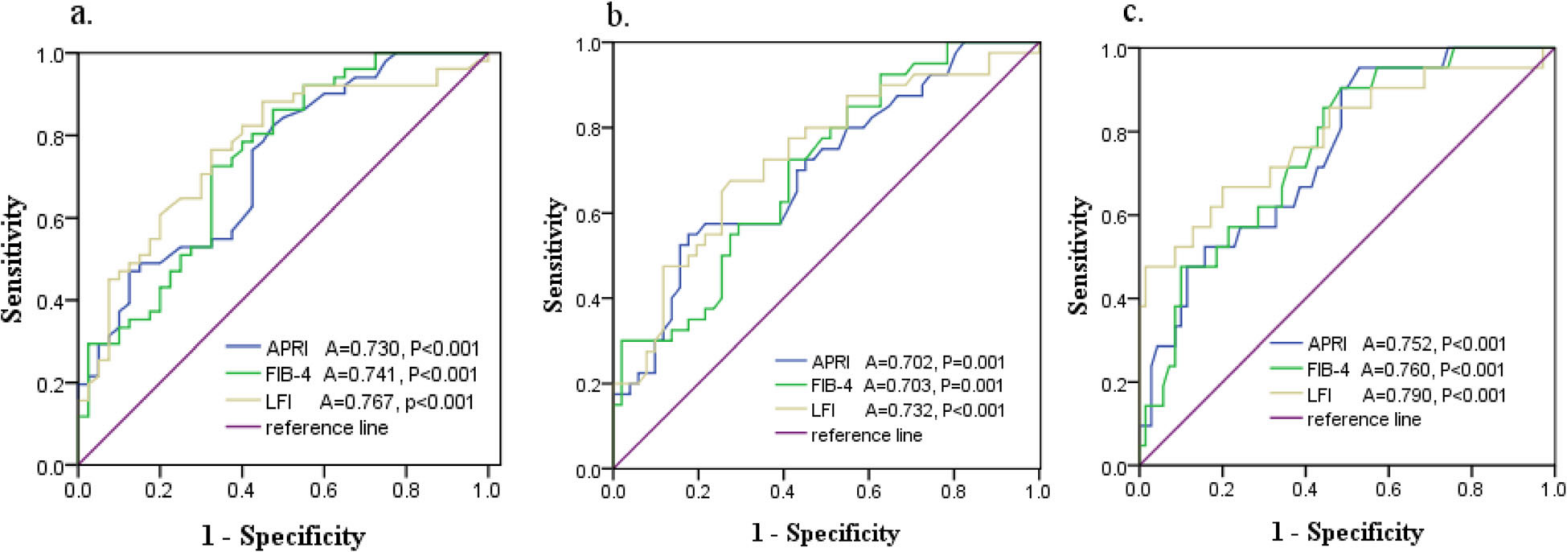
Receiver operating curves (ROC) of the APRI, FIB-4 and LFI for predicting significant LF (F ≥ F2) (**a**), advanced LF (F ≥ F3) (**b**) and liver cirrhosis (F ≥ F4) (**c**) APRI: aspartate aminotransferase-to-platelet ratio index, FIB-4: fibrosis index based on four factors, LFI: liver fibrosis index

Although the APRI, FIB-4 and LFI were able to predict significant LF (F ≥ F2) (Fig. 3a), advanced LF (F ≥ F3) (Fig. 3b) and liver cirrhosis (F ≥ F4) (Fig. 3c), the AUCs of the LFI were higher than those of the APRI or FIB-4. With significant LF (F ≥ F2) as a diagnostic criterion, the AUC of the LFI was 0.767, which was higher than those of the APRI and FIB-4, with a sensitivity of 81.6% and a specificity of 80.4%. The AUC of the LFI for diagnosing advanced liver fibrosis (*P* < 0.001) was significantly higher than those of the APRI and FIB-4. For predicting liver cirrhosis, the LFI was also superior to the APRI and FIB-4, with an AUC of 0.790, a sensitivity of 82.3% and specificity of 83.7%. The cut-off values of APRI for diagnose F > F1, F2, F3 were 0.58, 1.53, 2.07 respectively. The cut-off values of FIB-4 to predict F > F1, F2, F3 were 4.68, 5.76, 7.83. The LFI cut- off values to predict F > F1, F2, F3 were 2.61, 3.20, 3.92. The sensitivity and specificity of LFI cut-off values to predict the LF stages are better than APRI or FIB-4 to predict the stages. (Table 3).

**Table 3:** Diagnostic Accuracy of Different Methods for Prediction of Liver Fibrosis

	Optimized cut off value	Sensitivity (%)	Specificity (%)	AUC (95%CI)	*P* value of ROC contrast test
F0-F1 vs. F2-F4					
APRI	0.58	62.38	71.29	0.73 (0.63, 0.76)	< 0.001
FIB-4	4.68	60.63	65.56	0.74 (0.68, 0.80)	< 0.001
LFI	2.61	81.62	80.47	0.77 (0.69, 0.82)	< 0.001
F0-F2vs.F3-F4					
APRI	1.53	62.65	70.26	0.70 (0.63, 0.74)	< 0.001
FIB-4	5.76	64.48	63.19	0.70 (0.66, 0.71)	0.001
LFI	3.20	69.82	74.26	0.73 (0.65, 0.77)	< 0.001
F0-F3 vs. F4					
APRI	2.07	60.73	62. 68	0.75 (0.69, 0.84)	< 0.001
FIB-4	7.83	59.08	58.83	0.76 (0.66, 0.79)	< 0.001
LFI	3.92	82.29	83.71	0.79 (0.70, 0.88)	< 0.001

Optimized cut off value: were chosen by Youden Index which was the optimal combination of sensitivity and specificity, *APRI* aspartate aminotransferase-to-platelet ratio index, *FIB-4* fibrosis index based on four factors, *LFI* liver fibrosis index

## Discussion

Early diagnosis and accuracy measurement of the degree of LF or cirrhosis is essential not only for CHB patients making decisions to accept antiviral treatment as soon as possible, but also for controlling disease progression [6, 21, 28]. Liver biopsy remains the gold standard for assessing the stage of LF or cirrhosis, but this procedure is invasiveness, complication and may cause physical and mental discomfort [25]. Moreover, because of sampling errors, liver biopsy is susceptible to intraobserver and interobserver variability, and its poor reproducibility also need to be recognized again [8, 26]. Many scientists have focused on noninvasive techniques to identify LF grades or cirrhosis [4, 6, 8, 15, 16, 29]. The ideal noninvasive measurement of LF or cirrhosis, such as the APRI, FIB-4 and LFI, should be reproducible, reliable, simple, inexpensive and accurate for grading LF. Especially in resource-limited settings in China, the application of these noninvasive methods maybe reduces or replaces the need for liver biopsy in CHB patients [12, 13, 30].

The APRI, FIB-4 and LFI could differentiate the stage of LF in CHB patients because these indexes were significantly different for each stage of LF [28, 31]. As reported by Ren et al., the median of the APRI in stage F0, F1, F2, F3 and F4 LF was 0.21, 0.49, 0.49, 0.73 and 0.74, respectively, and that of the FIB-4 was 0.84, 1.09, 1.63, 1.59 and 2.03, respectively [31]. The APRI (*P* = 0.03) and FIB-4 (*P* < 0.001) were significantly different in different stages of fibrosis [31]. The APRI between stage F1 and F2 and between stage F3 and F4 were not significantly different. These results were partly consistent with the results in our study and another meta-analysis [32]. Though the APRI (*P* < 0.001) and FIB-4 (*P* < 0.001) were significantly different between stages of LF, no significant differences in the APRI and FIB-4 were found between stage F0 and F1 or between stage F3 and F4 in our study or another Egyptian study [28]. The APRI and FIB-4 may be influenced by many factors, such as age and the degree of liver inflammation. Age and AST in our study were higher than those reported by Ren et al. AS a fast, simple, safe and reliable noninvasive method recommended by the guidelines, [33] the LFI was not only significantly different between various ranges fibrosis stage, but also between each stage of fibrosis, which was proved by our study and other studies [20, 34]. The APRI, FIB-4 and LFI showed a significant correlation with the stages of fibrosis in our study and in some previous studies [20, 30, 34, 35]. As a noninvasive method, the LFI showed a better ability to differentiate the stage of liver fibrosis than did the APRI and FIB-4.

AUC < 0.7, the accuracy of identify is poor or fail; 0.7 ≤ AUC ≤ 0.9, the accuracy of identify is good; 0.9 < AUC ≤ 1, the accuracy of identify is excellent. The sensitivity or specificity of liver fibrosis measurements higher than 80% are applicable. Given that the APRI and FIB-4 are two readily available noninvasive methods for diagnosing LF, these methods have been recommended to determine the fibrosis stage in resource-limited countries by the WHO guidelines and by many other guidelines [1, 11–13]. Teshale et al. investigated the predictive ability of the APRI and FIB-4 for staging LF in a large cohort of CHB patients and found that the APRI and FIB-4 distinguished stage F2–F4 from stage F0-F1 with good sensitivity and specificity [30, 36]. The APRI and FIB-4 were also reported to have a high AUC for detecting significant fibrosis, advanced fibrosis and cirrhosis in 200 CHB patients in East Africa [28]. Hang et al. analysed four noninvasive tools, including the APRI and FIB-4, in a large Asian CHB patient cohort to diagnose significant fibrosis and obtained adjusted AUCs of 0.73 and 0.61 [35]. Zhang et al. analysed the APRI and FIB-4 in 1543 patients with HBV infection to predict cirrhosis and obtained adjusted AUCs of 0.71 and 0.79 in China [37]. In the 170 Chinese treatment-naive CHB patient cohort, the AUCs of the APRI for detecting significant fibrosis, advanced fibrosis and cirrhosis were 0.70, 0.63, and 0.71, respectively, and 0.76, 0.70, and 0.68, respectively for the FIB-4 [30]. In our study, the AUCs of the APRI for the prediction of significant LF, advanced LF and liver cirrhosis were 0.73, 0.70 and 0.75, respectively. However, a meta-analysis suggested that the APRI and FIB-4 could identify LF with only moderate sensitivity and accuracy in CHB patients and were not ideal replacement tests for liver biopsy [38, 39]. In our study, the APRI and FIB-4 could diagnose the LF stages with only moderate sensitivity and accuracy, and the results were consistent with previous studies [3, 28, 30, 35, 36]. As the LFI showed a better ability to differentiate the stage of LF than the APRI and FIB-4, the ability of the LFI to distinguish the liver fibrosis stage was analysed in our study. The AUC, sensitivity and specificity of the LFI for predicting mild, significant, advanced LF and cirrhosis were better than those of the APRI and FIB-4 in this study, which was consistent with the results of previous reports [9, 20]. The low (high sensitivity) and the high (high specificity) cut-off values were recommended by WHO guideline [11]:0.5 and 1.5 to distinguish F0–1 and F2–4,1.0 and 2.0 to differentiate F0–3 and F4 for APRI, 1.45 and 3.25 to distinguish F0–2 and F3–4 for FIB-4. In this study, a single cut-off value was chosen at maximizing of the sensitivity and specificity. The cut-off values of APRI was consistent with the values recommended by WHO guideline [11]. The single cut-off values for the diagnosis of significant LF and advanced LF and liver in this study was higher than those recommended by WHO guideline, but the sensitivity and specificity were only moderate. FIB-4 was not recommended for diagnosis of liver cirrhosis by WHO guideline. Though single cut-off value (7.83) could be used to diagnose liver cirrhosis, the sensitivity (59.08%) and specificity (58.83%) were not very well, in this study. For the diagnosis of F > F1, F2, F3 at the cut-off value 2.61, 3.20, 3.92 respectively, the sensitivity and specificity were better than those of APRI and FIB-4.

The LFI calculated using RTE with an HI VISION 900 ultrasound system had the highest predictive ability for identifying significant, advanced LF and cirrhosis among the studied noninvasive LF indexes in CHB patients in China, with higher sensitivity and accuracy than the APRI and FIB-4.

We acknowledge several limitations in our study. First, our patients were enrolled from a single referral centre, which may be have led to selection bias. Second, the LFI is influenced by several factors, such as patient cooperation with breathing, heart rate and selection of the ROI; thus, further studies with a larger sample population are needed. Third, the degree of fatty infiltration was not investigated. For the resource limitation, the LFI detected by RTE did not compared the results of Fibroscan in this study.

## Conclusions

In conclusion, the LFI, which was calculated using RTE with an HI VISION 900 ultrasound system, showed a better ability to differentiate the stage of LF than the APRI and FIB-4, especially between stages F0 and F1 and between stages F3 and F4. The LFI had a better predictive ability for identifying significant, advanced LF and cirrhosis than the APRI and FIB-4 in CHB patients in China, with higher sensitivity and accuracy.

## Data Availability

The datasets used and/or analyzed during this study are available from the corresponding author on reasonable request.

## Abbreviations

ALT: Alanine aminotransferase
APRI: Aspartate aminotransferase-to-platelet ratio index
AST: Aspartate aminotransferase
AUCs: Areas under the receiver operating characteristic curves
CHB: Chronic hepatitis B
FIB-4: Fibrosis index based on four factors
HBV: Hepatitis B virus
HCC: Hepatocellular carcinoma
LF: Liver fibrosis
LFI: Liver fibrosis index
PLT: Platelet count
ROI: Region of interest
RTE: Real-time ultrasound elastography
T-BIL: Total bilirubin
γ-GGT: γ-glutamyl transpeptidase
